# Optimizing Primary PCI Care for STEMI Patients: Key Messages From the SCAI Expert Consensus Statement

**DOI:** 10.1016/j.jscai.2024.102393

**Published:** 2024-10-07

**Authors:** Marc-André d’Entremont, Sanjit S. Jolly

**Affiliations:** a Population Health Research Institute, Hamilton, Ontario, Canada; b Hamilton Health Sciences, McMaster University, Hamilton, Ontario, Canada; c Centre Hospitalier Universitaire de Sherbrooke (CHUS), Sherbrooke, Quebec, Canada

While the recent European Society of Cardiology (ESC) acute coronary syndrome (ACS) guidelines provide a framework for overarching patient care objectives for all physicians, interventional cardiologists are likely to benefit from more practical and technical guidance to optimize primary percutaneous coronary intervention (PCI) in patients with ST-elevation myocardial infarction (STEMI) in the cardiac catheterization laboratory (CCL).^1^ In this issue of *JSCAI*, Tamis-Holland and colleagues^2^ report a consensus statement to advise interventional cardiologists in the primary PCI setting. The authors should be commended for completing this much-needed consensus statement and applauded for the breadth and depth of the document, ranging from technical details of PCI to the management of equipment in the CCL. Below, we highlight key take-home messages.

## Transradial first in STEMI

First, the authors emphasize the importance of a transradial-first approach for arterial access, given that access is one of the leading causes of morbidity and mortality during PCI. In an individual participant meta-analysis including 21,600 patients from randomized trials, radial access (compared with femoral access) was associated with a reduction in mortality (1.6% vs 2.1%; hazard ratio [HR], 0.77; 95% CI, 0.63-0.95; *P* = .012) and major bleeding (1.5% vs 2.7%; odds ratio [OR], 0.55; 95% CI, 0.45-0.67; *P* < .01).^3^ A transradial-first approach for PCI is gaining popularity in the United States as its use has increased from 23.9% in 2013 to 68.2% in 2022.^4^ The consensus document will positively impact this momentum toward using a transradial-first approach.

## Ultrasound guidance for femoral access

Second, the authors recommend using ultrasound guidance in cases where femoral access is chosen as the preferred route. An individual participant meta-analysis including 2441 participants randomized to ultrasound guidance vs no ultrasound guidance demonstrated a modest yet clinically relevant reduction in major vascular complications or bleeding at 30 days (2.8% vs 4.7%; OR, 0.61; 95% CI, 0.39-0.94; *P* = .026).^5^ Given the importance of reducing major access-site complications, new operators should perform at least 20 ultrasound-guided transfemoral access cases to decrease complication rates over non-ultrasound guidance, with additional accrued cases leading to increased proficiency.^6^ Furthermore, ultrasound machines should be available in all CCLs, just as for all other essential equipment mentioned in Table 1 of the paper. CCL staff should be expected to prepare the required ultrasound equipment as soon as transfemoral access is chosen.

## Intravascular imaging in STEMI

Third, the authors showcase the importance of routine intravascular imaging (IVI) while highlighting clinical scenarios where IVI is essential to optimize patient care. In the recent ESC Chronic Coronary Syndrome Guidelines, IVI was given a 1A recommendation for complex lesions, including left main, bifurcations, and long lesions.^7^ STEMI patients not infrequently have complex culprit lesions where IVI can usually be safely used to plan PCI once flow has been restored. The recent IVUS-ACS trial (*N* = 3505) was the first large trial to randomize intravascular ultrasound vs angiography alone for PCI exclusively in patients with ACS, of which 27.8% had STEMI.^8^ Target vessel failure occurred in 70/1753 patients in the IVUS group vs 128/1752 patients in the angiography group (HR, 0.55; 95% CI, 0.41-0.74; *P* < .01), with the effect being consistent in the STEMI subgroup (29/484 vs 56/489; HR, 0.54; 95% CI, 0.34-0.85) with no significant interaction (*P* = .26).^8^ Furthermore, the authors recommend the use of IVI in cases of stent thrombosis, stent failure, or myocardial infarction with nonobstructive coronary artery disease (MINOCA). An accurate diagnosis of stent failure causes such as underexpansion, neointimal hyperplasia, or neoatherosclerosis and the identification of plaque erosion, rupture, or healed plaque morphology in MINOCA likely optimizes subsequent percutaneous management strategies in the CCL.

## Cardiogenic shock

Fourth, the authors have updated the management of advanced cardiogenic shock in STEMI by considering microaxial flow pumps in the STEMI workflow based on the recent DanGer Shock randomized trial (*N* = 355).^9^ Compared with the standard of care, the microaxial flow pump was associated with a reduction in all-cause mortality at 180 days in patients with STEMI and SCAI stage C through E cardiogenic shock (82/179 [45.8%] vs 103/176 [58.5%]; HR, 0.74, 95% CI, 0.55-0.99; *P* = .04).^9^ Notably, the authors recommend right heart catheterization during the index primary PCI in patients with cardiogenic shock to better guide the need and the type of required mechanical circulatory support.

## Multivessel disease in STEMI

Fifth, the consensus statement highlights the importance of complete revascularization of the non-culprit lesions after primary PCI. The COMPLETE trial randomized 4041 STEMI patients with multivessel disease who had undergone successful culprit lesion PCI to a strategy of percutaneous complete revascularization of angiographically significant non-culprit lesions vs no further intervention. Complete revascularization was associated with a 26% relative risk reduction of cardiovascular death or myocardial infarction (158/2016 [7.8%] vs 213/2025 [10.5%]; HR, 0.74; 95% CI, 0.60-0.91; *P* = .004).^10^ Non-culprit lesion revascularization can be performed within the index hospitalization or as an outpatient in the months after the index procedure.

## Evidence gaps

The authors also identified evidence gaps where large randomized trials were needed. For example, routine manual thrombectomy during primary PCI compared with usual primary PCI has not been shown to decrease major adverse cardiovascular events but has been associated with an increased risk of stroke.^11^ A previous individual participant meta-analysis (*N* = 19,047) did suggest a numerical trend toward a benefit for cardiovascular death at 30 days in patients with TIMI thrombus grade ≥3 compared with usual care (2.5% vs 3.1%; HR, 0.80; 95% CI, 0.65-0.98; *P* = .03), although no significant interaction was found (*P* = .32).^11^ Randomized trials testing newer mechanical thrombectomy devices should be performed, as continuous suction may provide better thrombus clearance and a lower risk of thrombus embolization causing stroke.

## Conclusion

In summary, Tamis-Holland and colleagues have provided the interventional cardiology community with a practical framework for managing patients with STEMI in the CCL. Such work is instrumental in advancing the field, standardizing evidence-based CCL practices, and, most importantly, improving STEMI patient care.

## Declaration of competing interest

Marc-André d’Entremont is a Canadian Institutes of Health Research Canada Graduate Scholarship awardee. Sanjit Jolly reports receiving grants or contracts from Boston Scientific and payment or honoraria for lectures, presentations, speaker’s bureaus, manuscript writing, or educational events from Penumbra, Shockwave Medical, Teleflex, and Abiomed.

## Funding sources

This work was not supported by funding agencies in the public, commercial, or not-for-profit sectors.
